# The *fast* and the *slow* sides of cortisol's effects on emotional interference and sustained attention

**DOI:** 10.3389/fnins.2014.00268

**Published:** 2014-09-17

**Authors:** Florin Dolcos

**Affiliations:** Psychology Department, Neuroscience Program, and Beckman Institute for Advanced Science and Technology, University of Illinois at Urbana-ChampaignUrbana, IL, USA

**Keywords:** emotional distraction, emotional interference, stress hormones, brain imaging, emotion-cognition interactions, stress disorders

The paper by Henckens and colleagues is a very welcome addition to the literature on emotion-cognition interactions, in general, and on the impact of stress, in particular. By combining functional magnetic resonance imaging (fMRI) with carefully controlled cortisol administration, this study explored the neural substrates of time-dependent effects of stress hormones on attentional processing and emotion interference. Findings suggest a temporally fine-tuned cortisol's action, with an initial surge in vigilance that impairs *selective* attention (reflected in increased emotional interference), followed by a facilitation of *sustained* attention, seemingly contributing to the restoration of brain function following stress. These findings have important implications for understanding the stress hormones' effects on affective and cognitive processing in healthy functioning, and provide insights into possible mechanisms for stress-related disorders, such as post-traumatic stress disorder (PTSD).

Previous research provided evidence that stress hormones impact cognition and behavior (McEwen et al., 1995; de Kloet et al., 2005; Arnsten, 2009), with corticosteroids, in particular, having profound influences on both affective and cognitive functions (de Kloet et al., 1999; Erickson et al., 2003; Roozendaal et al., 2006b), as they can easily cross the blood–brain barrier and readily bind to receptors located in emotion (amygdala—AMY) and cognitive (hippocampus—HC and prefrontal cortex—PFC) processing brain regions (Lupien et al., 2007; Roozendaal et al., 2009). Recent evidence from research in rodents suggests that corticosteroids can induce rapid, non-genomic effects followed by slower, genomic effects that can impact cognitive functions in opposite and complementary ways (Karst et al., 2005; Wiegert et al., 2005). Traditionally, animal research has focused on the effects of corticosteroids on HC, where corticosteroids' genomic effects have been known for decades to *suppress* neuronal excitability (Joëls and de Kloet, 1989; Kerr et al., 1989) and long-term potentiation (LTP) (Pavlides et al., 1995; Wiegert et al., 2005), the alleged neurobiological substrate of memory formation (Martin and Morris, 2002). However, recent findings indicated that corticosteroids *increase* hippocampal neuronal excitability (Karst et al., 2005) and LTP (Korz and Frey, 2003; Wiegert et al., 2006) in a rapid, non-genomic fashion, but only when present around the time when LTP is induced. Similar excitatory rapid effects have been also observed in AMY (Karst et al., 2010).

Despite evidence of time-dependent effects of corticosteroids in rodents, temporal dynamic effects of cortisol on affective and cognitive functions have only recently started to be investigated in humans (Henckens et al., 2010, 2011, 2012; Hermans et al., 2014). Henckens et al. (2012) investigated the time-dependent impact of cortisol on the neural correlates of attentional processing by using a randomized, double-blind, placebo-controlled approach, involving the following 3 groups: (1) *placebo* (receiving placebo 270 and 60 min before the task), (2) *rapid cortisol* (receiving placebo and hydrocortisone, 270 and 60 min before task, respectively), and (3) *slow cortisol* (receiving hydrocortisone and placebo 270 and 60 min before the task, respectively).

Functional MRI data were recorded while participants performed an emotional distraction task, which allowed examination of both selective and sustained attention. Selective attention was measured as the difference in interference produced by emotional compared to neutral distraction, whereas sustained attention was reflected in the overall performance in trials with both emotional and neutral distraction. Thus, compared to previous studies, the approach used by Henckens and colleagues has the clear advantage of allowing examination of corticosteroid effects in a time-dependent manner on different types of attentional processing and on emotion processing. First, results indicated that the rapid effects of corticosteroids were associated with increased bottom-up/stimulus-driven attentional processing, which caused impaired selective attention (as reflected in increased emotional interference), associated with increased activity in the AMY and increased AMY-PFC connectivity while processing aversive relative to neutral distraction. These findings from the *fast* cortisol group suggest that the rapid corticosteroid effects cause stimulus-driven behavior, and can contribute, together with those of catecholamines, to a state of hypervigilance (Roozendaal et al., 2006a; Joëls and Baram, 2009). Second, the slow effects of corticosteroids modulated the neural correlates of sustained attention, by reducing bottom-up processing. Specifically, the *slow* cortisol group showed reduced activation in visual brain regions linked to sustained attentional processing, as well as reduced negative connectivity between activity in the AMY and insula. These findings suggest that the slow corticosteroid effects might counteract the rapid effects by reducing automatic visual/stimulus-driven processing and engaging more controlled processing to restore brain functions following stress.

Overall, these findings indicate that corticosteroids influence brain function in a time-dependent manner, affecting activity and connectivity of visual, emotional, and cognitive processing brain regions in an opposite manner, in order to serve adaptation to changing environmental demands. Thereby, this study proposes a more adaptive view on the impact of cortisol on attention and emotion according to the temporal profile of action, with an initial effect optimizing detection of potential threat at the cost of impaired cognitive processing, and a delayed effect normalizing cognitive brain functions following stress (see also Joëls et al., 2011; Hermans et al., 2014). Of note, while these effects might allow for optimal responding to stressful situations and subsequent recovery in healthy individuals, they are likely impaired in PTSD, which is characterized by a continuous state of hyper vigilance (Dolcos, 2013). These findings highlight the importance of timing in the effects of stress hormones, as a critical factor to take into account in future studies, and point to a more adaptive view on the effects of emotion (or stress) on cognition, depending on the circumstances. The importance of considering opposing effects of emotion on cognition is also reflected in the success of the Special Research Topic that this report is part of Dolcos et al. (2012–2014), which attracted numerous outstanding contributions regarding the mechanisms of emotion-cognition interactions. I anticipate that this is only the beginning of what is yet to come in the field, and the paper by Henckens and colleagues is riding right at the top of this exciting emerging research wave!

## Conflict of interest statement

The author declares that the research was conducted in the absence of any commercial or financial relationships that could be construed as a potential conflict of interest.
